# Characterization of Anthocyanins in *Perilla frutescens* var. *acuta* Extract by Advanced UPLC-ESI-IT-TOF-MS*^n^* Method and Their Anticancer Bioactivity

**DOI:** 10.3390/molecules20059155

**Published:** 2015-05-19

**Authors:** Yan-Kang He, You-Yuan Yao, Ya-Ning Chang

**Affiliations:** Department of Applied Biology, College of Bioengineering, East China University of Science and Technology, Shanghai 200237, China; E-Mails: heyankang@live.cn (Y.-K.H.); 030121237@mail.ecust.edu.cn (Y.-Y.Y.)

**Keywords:** *Perilla frutescens* var. *acuta*, anthocyanins, ESI-IT-TOF-MS*^n^*, Hela cells, apoptosis

## Abstract

The anthocyanin extract from a domestic Perilla cultivar (*Perilla frutescens* var. *acuta*) were isolated and characterized with high mass accuracy and multi-dimensional fragmentation by means of ultra-performance liquid chromatography (UPLC) and electrospray ionization-ion trap-time of flight mass spectrometry analysis (ESI-IT-TOF-MS*^n^*). The new developed and applied LC-MS method focused on in-depth screening of anthocyanin compounds with similar structures which also provided a new approach of anthocyanin characterization without the use of external standards. Selective detection of interested anthocyanins was achieved utilizing extracted ion chromatogram (EIC) analysis, while MS*^n^* spectra were recorded to allow identification of the anthocyanin based on characteristic fragmentation patterns. Seven anthocyanins including one feruloyl (Cyanidin 3-*O*-feruloylglucoside-5-*O*-glucoside), two caffeoyl (Cyanidin 3-*O*-caffeoylglucoside-5-*O*-glucoside, Cyanidin 3-*O*-caffeoylglucoside-5-*O*-malonylglucoside) and four coumaroyl substituted anthocyanins (*Cis*-shisonin, Malonyl-*cis*-shisonin, Shisonin, and Malonyl-shisonin) were identified. Annexin-V FITC/PI flow cytometric assay was performed to analyze the influence of anthocyanin extract of *P. frutescens* var. *acuta* on cell apoptosis. The results suggested that Perilla anthocyanins can induce Hela cell apoptosis by a dose dependent manner.

## 1. Introduction

**Figure 1:**
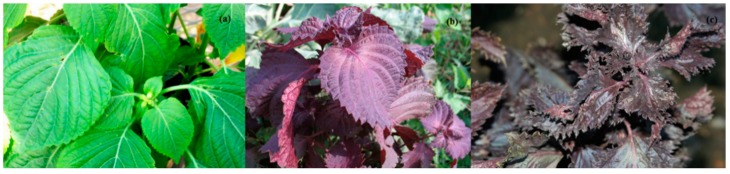
Three natural varieties of *Perilla frutescens* (**a**) *P. frutescens* var. *frutescens*; (**b**) *P. frutescens* var acuta; (**c**) *P. frutescens* var crispa.

Perilla (*Perilla frutescens* (L.) Britt.) is an annual herbaceous plants native to Eastern Asia. It is well-known in Chinese medicine and has a long history of cultivation in China as a source of medicine and spices. Among the common varieties of *P. frutescens*, three are widely used by local people which are generally known as var. *frutescens*, var. *crispa* and var*. acuta* (Figure 1)*.* Seeds of *P. frutescens* var. *frutescens* are considered as a rich oil source and its leaves are edible and also used as a preservative in the food industry. Similarly, *P. frutescens* var. *crispa* are also used as fresh vegetables by people from the Far East, but more research is focused on its medicinal properties [1,2]. *P. frutescens* var. *acuta* are particularly known for prevention and treatment of seafood poisoning by fishes or crabs contaminated with bacteria that produce various toxins, and are also shown to have other bio-activities like anti-oxidative, anti-inflammatory and anti-allergic abilities [3,4,5].

Different chemotypes have been found within varieties of *P. frutescens*, which lead to the diversity of leaf and stem colors, indicating the existence of a great range of anthocyanins in these plants. Related researches on anthocyanin contents in var. c*rispa* have shown that the red color was given by the presence of a cyanidin-type anthocyanin, cyanidin 3-(6-*p*-coumaroylglucoside)-5-malonylglucoside, malonyl-shisonin [6], and other less abundant anthocyanin compounds that accumulate in the epidermal cells of leaves and stems of *P. frutescens* [7]. On the other hand, biological activities like antioxidant effects have been widely studied for *P. frutescens* varieties which indicated their medical potentials [4,8]. However, only limited studies were available for anthocyanin constituents and anticancer activity of *P. frutescens* var. *acuta*, though anthocyanins from other plant materials have exhibited a wide range of anticancer properties including antioxidant effects, anti-cell proliferation, induction of apoptosis, anti-inflammatory effects, anti-angiogenesis and *et al*. [9,10]. It is possible that Perilla anthocyanins have anticancer potential.

In the present study, therefore, an electrospray ionization-ion trap-time of flight mass spectrometer equipped with an ultra-performance liquid chromatograph (UPLC-ESI-IT-TOF-MS*^n^*) was used to identifying anthocyanin compounds in leaves of *P. frutescens* var. *acuta*. UPLC-ESI-IT-TOF-MS are complementary alternatives for characterizing isomers or anthocyanins with similar structures which show similar retention times and spectroscopic characteristics on HPLC-DAD analysis [11]. These soft ionization, multi stage fragmentation and high resolution techniques provide mass spectra with intact molecular ion and high accuracy of full-fragment spectra making it possible to define the distribution of anthocyanins in complex matrices. For now, to our knowledge, high mass accuracy and MS*^n^* measurements of anthocyanins appear to be limited in literature. Thus, one of the objectives of this study was to develop an advanced LC-MS system to confirm the identity of anthocyanins which have similar structures without the use of reference standards*.* Another objective was to investigate the induction ability on cell apoptosis of its extract according to DAPI staining and Annexin-V FITC/PI flow cytometric assay since it has been reported that anthocyanins can induce apoptosis through both intrinsic (mitochondrial) and extrinsic (FAS) pathways [9].

## 2. Results and Discussion

### 2.1. Total Anthocyanin Content (TAC)

The total monomeric anthocyanin content in the leaves of *P. frutescens* var. *acuta* was 52.5 mg/100 g dry weight, expressed as cyanidin-3-*O*-glucoside equivalents and calculated as the mean value of three measurements and the standard deviation. In a previous report, total anthocyanin content of two var. *frutescens* cultivars from different regions of China are 1.6 mg/g (dry matter) and 1.8 mg/g (dry matter), respectively, whereas two var. *crispa* cultivars from China and Japan are 0.7 mg/g (dry matter) and 1.6 mg/g (dry matter), respectively. Apart from the difference between *P. frutescens* varieties, different levels of structural anthocyanin gene expression related to cultivation environment and harvest time may explain this difference of anthocyanin content [1].

### 2.2. UPLC-ESI-IT-TOF-MS^n^ Analysis

Although there are various reports of using conventional HPLC with DAD detection at 520 nm for the determination of anthocyanins in complex plant extracts [11], such methods cannot produce very accurate results, especially for those anthocyanins with similar structures. Despite the effect of matrix, the anthocyanins with similar structures may easily elute together as an unresolved broad peak, which increases the difficulty of qualitative analysis. On the other hand, LC-MS analysis permits more accurate results and provides benefits such as increased sensitivity and structural information compared to LC-DAD [12]. Moreover, application of ESI-IT-TOF effectively simplifies the procedure of characterization and structure elucidation and makes it possible for the identification of anthocyanins without the aid of standards, enabling the establishment of anthocyanin profile and structure through high accuracy molecular mass determination and multistage fragmentation of the ions of interest in merely a single assay.

As can be seen, seven anthocyanins were detected. Table 1 shows the detailed information obtained by UPLC-TOF-MS, including retention time, maximum absorption (λ_max_), theoretical and measured mass signals of molecular ion ([M]^+^), accurate mass, error associated with the accurate mass (ppm) and the molecule formula, while Table 2 summarizes the MS*^n^* data collected by the QIT procedure and subsequent fragment assignments. As mentioned above, the higher accuracy and sensitivity of the TOF system provided quite a sharp profile from which the main anthocyanins can be easily identified through extracted ion chromatograms (EIC). Moreover, when analysed by MS*^n^*, anthocyanins rapidly lose the sugar moiety, and the molecular ion [M]^+^ occurs together with the somewhat less-intense fragment ions [M-sugar moiety]^+^ or [M-sugar moiety-organic acid]^+^. These fragmentation features may provide extra information on the structure of the compound which can be useful to the identification of the anthocyanin. However, on some occasions the aglycone fragment was the only one present in the MS*^n^* spectra, and this made the TOF function of accurate mass measurement (less than 5 ppm error in the IT-TOF-MS system) crucial to the qualitative analysis. In addition, the abundances of the isotope peaks of the molecular ions ([M]^+^) were also monitored to confirm the identities of compounds. Therefore, UPLC-ESI-IT-TOF-MS*^n^* experiment which simultaneously performs TOF and MS*^n^* function has its unique advantages in the field of natural product characterization, especially anthocyanin identification.

**Table 1:** Profile of anthocyanin compounds from *P. frutescens* var. *acuta* determined by LC-ESI-IT-TOF-MS*^n^.*

Peak No.	Compound Assigned	*Rt* (min) ^a^	λ_max_ (nm)	Measured Signal (*m/z*)	Therotical Mass (*m/z*)	Error (ppm)	Molecular Weight (m/z)	Molecule Formula
1	*Cis*-shisonin	6.27	283, 315, 528	757.1965	757.1974	1.19	757.1980	C_36_H_37_O_18_^+^
2	Cyanidin 3-*O*-caffeoylglucoside- 5-*O*-glucoside	6.35	283, 318, 525	773.1932	773.1924	1.03	773.1929	C_36_H_37_O_19_^+^
3	Cyanidin 3-*O*-caffeoylglucoside- 5-*O*-malonylglucoside	7.42	282, 317, 525	859.1926	859.1927	1.16	859.1933	C_39_H_39_O_22_^+^
4	Malonyl-*cis*-shisonin	7.53	282, 313, 527	843.1965	843.1978	1.54	843.1984	C_39_H_39_O_21_^+^
5	Shisonin	7.76	284, 315, 525	757.1973	757.1974	0.26	757.1980	C_36_H_37_O_18_^+^
6	Cyanidin 3-*O*-feruloylglucoside- 5-*O*-glucoside	8.18	283, 316, 523	787.2097	787.2080	2.16	787.2086	C_37_H_39_O_19_^+^
7	Malonyl-shisonin	8.91	283, 312, 528	843.1952	843.1978	3.08	843.1984	C_39_H_39_O_21_^+^

^a^ Retention time was recorded according to the peaks from extracted ion chromatogram (EIC).

**Table 2:** The fragment ions and types of seven anthocyanins originated from the precursor ions [M]^+^ in positive ion (PI) mode from *P. frutescens* var. *acuta*.

Anthocyanins	MS*^n^* Fragment Ions in Positive Ion Mode (*m*/*z*) (Fragment Type) ^a,b^		
	MS^1^	MS^2^	MS^3^
*Cis*-shisonin	757.1965 [M]^+^	595.1422 [M-Glc]^+^ 287.0556 [Cy]^+^ 449.1002 [M-Glc-Cou]^+^	287.0541 [Cy]^+^
Cyanidin 3-*O*-caffeoylglucoside- 5-*O*-glucoside	773.1932 [M]^+^	611.1394 [M-Glc]^+^ 287.0549 [Cy]^+^ 449.1051 [M-Glc-Caf]^+^	287.0543 [Cy]^+^
Cyanidin 3-*O*-caffeoylglucoside- 5-*O*-malonylglucoside	859.1926 [M]^+^	287.0560 [Cy]^+^ 535.1085 [M-Glc-Caf]^+^ 611.1397 [M-Glc-Mal]^+^ 491.1186 [M-Glc-Caf-CO_2_]^+^ 815.2015 [M-CO_2_]^+^ 449.1007 [M-Glc-Mal-Caf]^+^	287.0552 [Cy]^+^ 491.1178 [M-Glc-Caf-CO_2_]^+^ 449.1007 [M-Glc-Mal-Caf]^+^
Malonyl-*cis*-shisonin	843.1965 [M]^+^	595.1440 [M-Glc-Mal]^+^ 535.1077 [M-Glc-Cou]^+^ 287.0560 [Cy]^+^ 491.1178 [M-Glc-Cou-CO_2_]^+^ 799.2107 [M-CO_2_]^+^	287.0550 [Cy]^+^ 491.1175 [M-Glc-Cou-CO_2_]^+^ 449.1033 [M-Glc-Mal-Cou]^+^
Shisonin	757.1973 [M]^+^	595.1444 [M-Glc]^+^ 287.0537 [Cy]^+^ 449.1059 [M-Glc-Cou]^+^	287.0541 [Cy]^+^
Cyanidin 3-*O*-feruloylglucoside- 5-*O*-glucoside	787.2097 [M]^+^	625.1547 [M-Glc]^+^ 287.0523 [Cy]^+^ 449.1104 [M-Glc-Fer]^+^	287.0539 [Cy]^+^
Malonyl-shisonin	843.1952 [M]^+^	595.1459 [M-Glc-Mal]^+^ 535.1095 [M-Glc-Cou]^+^ 287.0543 [Cy]^+^ 491.1181 [M-Glc-Cou-CO_2_]^+^ 799.2019 [M-CO_2_]^+^	287.0551 [Cy]^+^ 491.1148 [M-Glc-Cou-CO_2_]^+^ 449.1062 [M-Glc-Mal-Cou]^+^

Abbreviations. Cy, cyanidin; Glc, glucoside; Cou, *p*-coumaroyl moiety; Caf, caffeoyl moiety; Fer, feruloyl moiety; Mal, malonyl moiety; ^a^ Precursor ions in MS^2^ were underlined; ^b^ Fragments were listed in the order of abundance.

#### 2.2.1. Extracted Ion Chromatogram (EIC) Analysis

**Figure 2:**
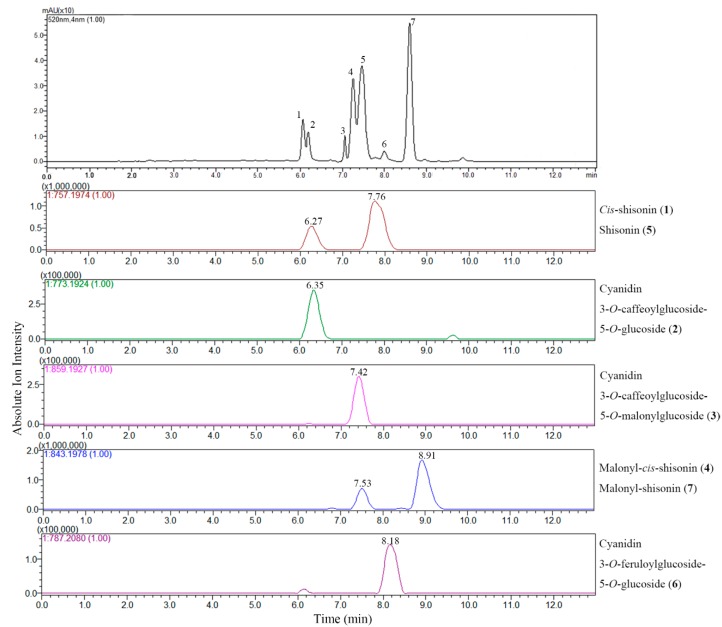
LC-DAD chromatogram at 520 nm and extracted ion chromatograms (EIC) of LC-ESI-IT-TOF-MS analysis of anthocyanins in *P. frutescens* var. *acuta* extract.

The LC-DAD chromatogram and extracted ion chromatograms (EIC) of the extract of *P. frutescens* var. *acuta* subjected to positive ion ESI-IT-TOF analysis are shown in Figure 2. EICs were obtained at each *m*/*z* value corresponding to the theoretical mass of the anthocyanins that were tentatively assigned in order to identify the compound clearly. Cyanidin 3-*O*-caffeoylglucoside-5-*O*-glucoside (**2**) ([M]^+^, *m*/*z* 773.1924), Cyanidin 3-*O*-caffeoylglucoside-5-*O*-malonylglucoside (**3**) ([M]^+^, *m*/*z* 859.1927), Cyanidin 3-*O*-feruloylglucoside-5-*O*-glucoside (**6**) ([M]^+^, *m*/*z* 787.2080) showed only one peak in EICs, while the EIC for *m*/*z* 757.1974 showed two peaks, one at *R_t_* 6.27 min and the other at *R_t_* 7.76 min, both presenting the same fragmentation patterns, suggesting the existence of isomers of shisonin. According to a previous report [6], the one at *R_t_* 6.27 min was assigned as *cis*-shisonin (**1**) and the other was identified as shisonin (**5**), which were distinguished by their ion abundance and retention time. The EIC for *m*/*z* 843.1978 also showed two peaks, one with relatively weak intensity at *R_t_* 7.53 min suggesting to be a *cis-* isomer of malonyl-*cis-*shisonin (**4**), and the other at *R_t_* 8.91 min exhibiting higher intensity thus confirming its identity as a *tran*- isomer of malonyl-shisonin (**7**) [7]. These two pairs of isomers are the main anthocyanin components existed in the *P. frutescens* which is in good agreement with previous literature [6,7]. Although accurate mass measurements alone could not differentiate between *cis-* and *trans-* isomers (which have identical molecular formula), the EIC analysis easily separated the two isomers, which provided another option for reliable data about elution order and abundance of anthocyanins when the LC chromatogram cannot give sufficient information.

**Figure 3:**
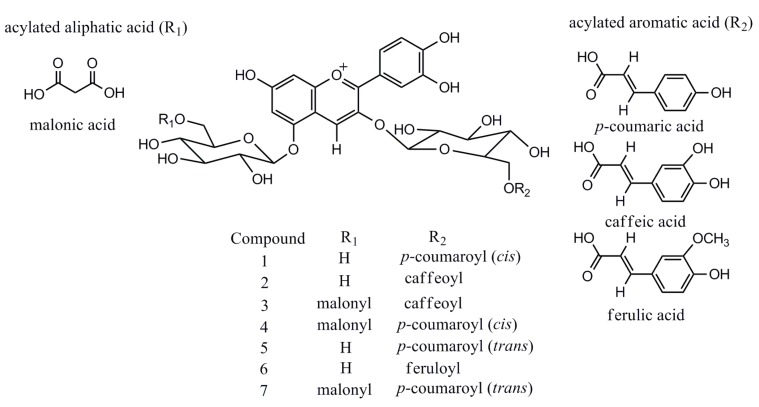
Structures of major anthocyanins found in *P. frutescens* var. *acuta*.

LC-MS in full scan mode has been used extensively for screening of anthocyanin profiles of *P. frutescens*, even though compound identification is often tentatively based on elution order, molecular ion information and fragmentation features only [6,7]. However, the EIC analysis selectively improves the sensitivity of targeted analytes, excluding the interference from other phenolic compounds in TIC, which, in this regard, is highly effective in the characterization of interested bioactive compounds in complex matrices of plant extracts. All the anthocyanins characterized in *P. frutescens* var. *acuta* extract are reported in Figure 3. Further literature searches confirm the identity of the six compounds except for a new found feruloyl substituted anthocyanin (**6**), which only showed a trace amount presence [6]. Although these six anthocyanins were previously identified by [6,7,8], we have documented for the first time the existence in *P. frutescens* var. *acuta*. On the other hand, sugar residues of anthocyanins can be easily acylated with organic acids, and common acylating agents include cinnamic acids such as caffeic, *p-*coumaric, ferulic and sinapic acid [13]. Among Perilla anthocyanins, to the best of our knowledge, coumaric and caffeic acid are common substitution moieties [6,7]. However, this is the first time that the existence of ferulic acid moiety has been identified in *P. frutescens*.

#### 2.2.2. Fragmentation Patterns of Perilla Anthocyanins

MS*^n^* function was known for its capability of successive fragmentation steps which can provide more structural information on the compound. Since few reference standards for anthocyanins are available (in particular, no standards are available for derived anthocyanins in plant extracts), those anthocyanins which were tentatively assigned through accurate mass measurement can only be further identified by analyzing their fragmentation behaviors in MS^2^ and MS^3^ spectra and by referring to previous reports.

**Figure 4:**
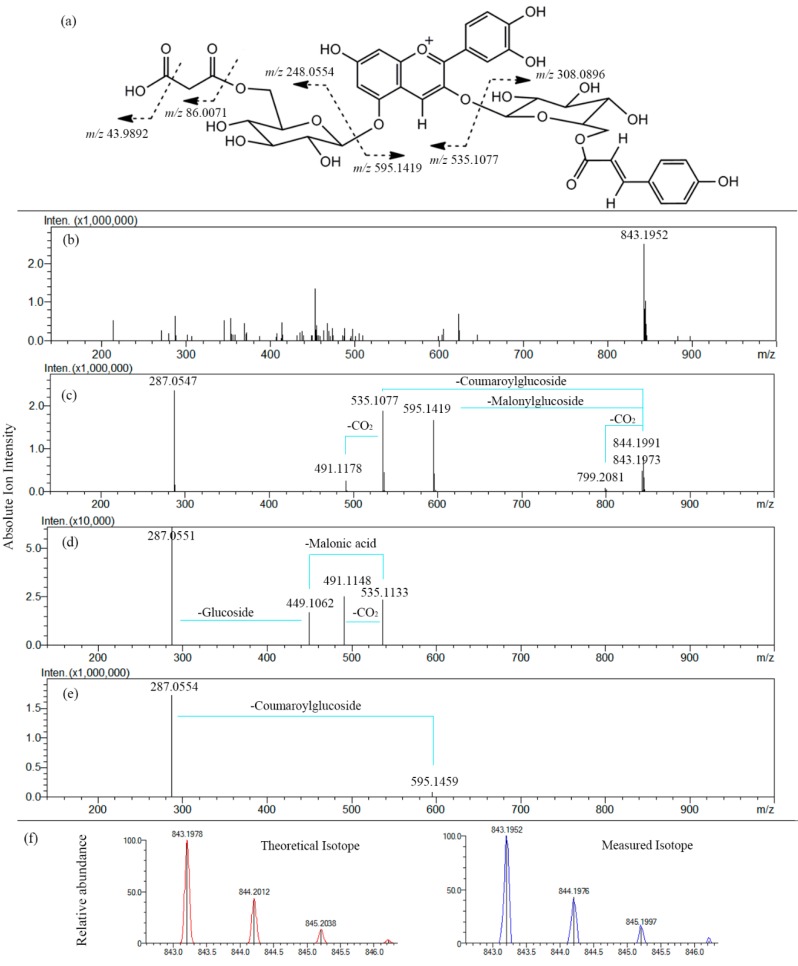
ESI-MS^3^ analysis of malonyl-shisonin in *P. frutescens* var. *acuta* extract. Positive ion mode. Collision Energy 25%. (**a**) Fragmentation features; (**b**) MS^1^ scan spectrum; (**c**) MS^2^ spectrum with fragments assigned; (**d**) MS^3^ spectrum (precursor ion *m*/*z* 595.1419); (**e**) MS^3^ spectrum (precursor ion *m*/*z* 535.1077); (**f**) Patterns of isotopes of the molecular ion *m*/*z* 843.1952.

The ESI-MS*^n^* spectrum of a typical anthocyanin (malonyl-shisonin) (**7**) is shown in Figure 4. The TOF-MS^1^ scan data showed that the molecular ion was *m*/*z* 843.1952 ([M]^+^) which was calculated as a molecular formula of C_39_H_39_O_21_^+^. The measured mass value obtained for this molecular ion was very close to the theoretical value (*m*/*z* 843.1978) of C_39_H_39_O_21_^+^ with an error of −3.08 ppm. In comparison with the theoretical values, the obtained ratios and abundance of the isotope ions for [M]^+^ was also analyzed to further confirm the validity of the molecular ion, which, as a result, exactly matches with the theoretical values with an Iso score of 85.72 (100). Moreover, The MS^2^ fragmentation of the molecular ion ([M]^+^, *m*/*z* 843.1952) produced several fragment ions: the flavylium cation of cyanidin ([Cy]^+^, *m*/*z* 287.0547) was confirmed, based on the mass accuracy −1.05 ppm error, as compared with the theoretical mass (*m*/*z* 287.0550, C_15_H_11_O_6_^+^). Signals for neutral loss of a malonylglucoside moiety, [M-Glc-Mal]^+^ (*m*/*z* 595.1419) and loss of a coumaroylglucoside moiety, [M-Glc-Cou]^+^ (*m*/*z* 535.1077) were also observed as the main fragment ions, corresponding to cyanidin 3-*O-*coumaroylglucoside and cyanidin 5-*O*-malonylglucoside, respectively.

In addition to molecular ion and fragment ion assignment, high mass accuracy was also used in analyzing the neutral losses of fragment ions. For example, In MS^2^ spectra, the loss of CO_2_ (43.9892 Da) can be differentiated from the loss of C_2_H_4_O (44.0262 Da) by determining the accuracy between the observed signal (43.9892 Da) and the theoretical monoisotopic mass.

The two most abundant fragment ions (*m*/*z* 595.1419, *m*/*z* 535.1077) in the MS^2^ spectra were then selected as precursor ions to trigger CID for further exploration of the correlation between fragmentation patterns and structural features in MS^3^ spectra. However, not many new fragment ions were found in MS^3^. Signals at *m*/*z* 449.1062 are believed to be [M-Glc-Mal-Cou]^+^, corresponding to cyanidin 5-*O*-glucoside which was due to the loss of the entire malonyl moiety.

The observed fragmentation patterns confirmed the known structure of malonyl-shisonin and are consistent with that reported by [6,7,14]. The published data of MS*^n^* refers to a fragmentation mechanism that cleavage occurs at C-O bond between aglycone and glucoside in positive ion mode. In MS^2^ mode, signals corresponding to the product ions were compatible with the combination of the independent losses of the glucose and acylated glucose moieties, in agreement with previously reported data obtained from various *P. frutescens* varieties [6,7].The acylated aromatic acid (*p*-coumaric acid, caffeic acid and ferulic acid) was stable and no fragment signals were discovered, while the acylated aliphatic acid (malonic acid) easily yielded neutral fragment losses (CO_2_) or entire cleavage from the glucoside. Similar fragmentation patterns were observed for other anthocyanins detected in *P. frutescens* var. *acuta*.

### 2.3. Cell Morphology Changes during Apoptosis

**Figure 5:**
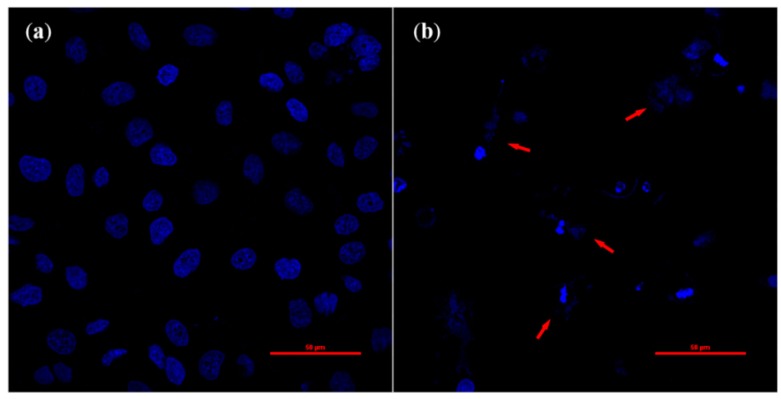
Effects of Perilla anthocyanins on cell apoptosis induction (600×) after DAPI staining (**a**) Control (**b**) 300 μg/mL.

It has been reported that the induction of apoptosis is one of the major mechanisms of anthocyanin-mediated cell death [15,16,17]. Since observation of cell morphology is the most direct way to know whether the cells are undergoing apoptosis, a traditional fluorescence staining method was used. Our results showed that with the increase of anthocyanin concentrations, apoptosis obviously increased in a dose dependent manner and the classical apoptotic cells were observed in 300 μg/mL anthocyanin-treated cells. Results of multiple concentrations of anthocyanin-treated cells are given in the Supplementary Material. Characteristic morphological changes of apoptosis from cell shrinkage and membrane blabbing to the formation of apoptotic bodies are shown in Figure 5. These results suggest that Perilla anthocyanins (300 μg/mL) can induce apoptosis in Hela cells after 24 h treatment.

### 2.4. Cell Apoptosis Detected by Annexin V-FITC/PI Assay

In apoptotic cells, the membrane phospholipid phosphatidyserine (PS) is translocated from inner to the outer leaflet of the plasma membrane, thereby exposing PS to the external cellular environment. Annexin V is a phospholipid-binding protein that has a high affinity for PS; thus, it can serve as a sensitive probe for cytometric analysis of cells that are undergoing apoptosis. Propidium iodide (PI) was used with FITC Annexin V to allow the investigation of early apoptotic cells (PI negative, FITC Annexin V positive).

**Figure 6:**
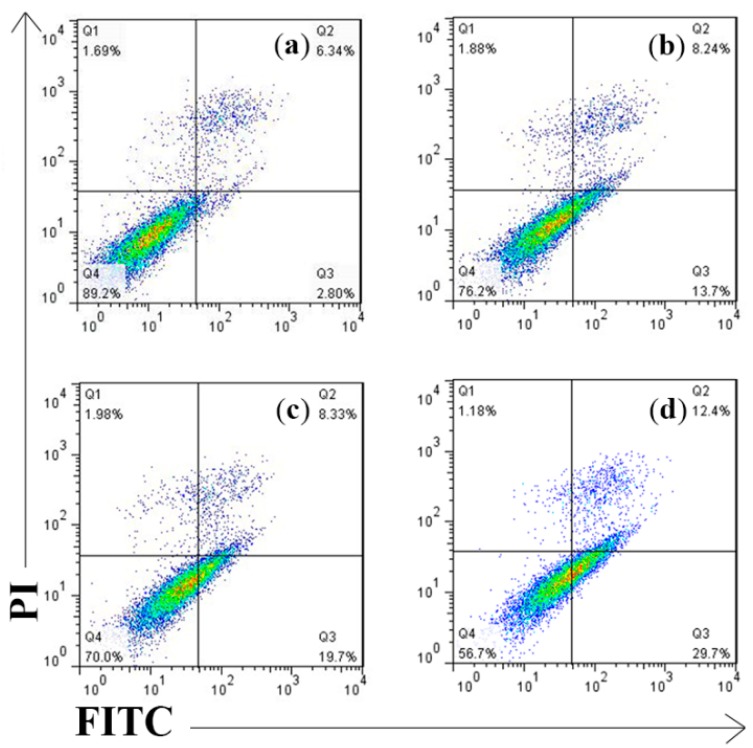
Measurement of Perilla anthocyanins on apoptotic rates by Annexin V FITC/PI analysis (**a**) Control (**b**) 200 μg/mL (**c**) 250 μg/mL (**d**) 300 μg/mL.

Flow cytometric analysis confirmed that the number of AV and AV/PI positive cells significantly increased in 300 μg/mL anthocyanin-treated groups at 12 h treatment. As been shown in Figure 6, the Q1(%) was 1.18% and the Q3(%) was 29.7%; thus, the respective apoptosis rates [Q2(%) + Q4(%)] were 42.1% indicating its high efficiency against Hela cells. Also, 300 μg/mL anthocyanin had a higher apoptotic ratio compared to 200 μg/mL (21.9%) and 250 μg/mL (28.0%) groups showing certain dose dependency.

## 3. Experimental Section

### 3.1. Plant Material

Perilla (*Perilla frutescens* var. *acuta*) leaves with red-green fresh leaf color were collected during September from wild in Longquan area (Zhejiang Province, China) (119°6′ east, 28°01′ north). Longquan area enjoys a comfortable subtropical monsoon climate featuring precipitation, full sunshine and four distinct seasons. Approximately 20 bunches were collected. Roots and rhizomes were removed subsequently. The fresh leaves once harvested were immediately packed with plastic sample containers in an ice box and delivered to our laboratory by overnight courier to prevent deterioration.

### 3.2. Reagents

All HPLC and LC-MS grade chemicals, including methanol and acetonitrile were purchased from Merck (Darmstadt, Germany). Ethanol, hydrochloric acid (HCl), acetic acid, formic acid and poly-sery HC-C18 SPE cartridges were purchased from ANPEL (shanghai, China). Hyclone fetal bovine serums (FBS), Dulbecco’s Modified Eagle Medium (DMEM), penicillin/streptomycin solution, dimethylsulfoxide (DMSO) were purchased from Thermo Fisher (Waltham, MA, USA). Trypsin-EDTA was obtained from Gibco (Invitrogen, Carlsbad, CA, USA). DAPI (4′,6-diamidine-2′-phenylindole) were purchased from Sigma-Aldrich (St. Louis, MO, USA). Amberlite XAD-7 was obtained from ANLAND (Shanghai, China). Annexin V-FITC/PI Apoptosis Detection Kit was purchased from BD (San Jose, CA, USA).

### 3.3. Cell Lines

The human cervix Adenocarcinoma Hela cell line was provided by the Department of applied biology, College of bioengineering, East China University of Science and Technology, Shanghai, China. The cells were cultured in Dulbecco’s Modified Eagle Medium (DMEM) supplemented with 10% heat-inactivated fetal bovine serum (FBS) and 1% penicillin/streptomycin antibiotics solution at 37 °C in a humidified atmosphere of 5% CO_2_.

### 3.4. Extraction and Isolation

Anthocyanins were extracted according to the method described by Zou *et al.* [18], with some modifications. Frozen dried Perilla leaves were ground to powder by a mortar and pestle. Then, powdery material (20 g) was extracted twice with 1000 mL 45% ethanol (*v*/*v*) in water (6% acetic acid). The extraction temperature was fixed at 45 °C and the extraction time was 60 min. Magnetic stirring was used during the extraction to improve solid-liquid exchange. The supernatant was concentrated in a rotary evaporator at 40 °C and then subjected to Amberlite XAD-7 column chromatography (2.5 × 50 cm). The column was washed with 400 mL of water, and elution of the anthocyanins was carried out with 400 mL of 95% ethanol. The eluate was concentrated under reduced pressure at 40 °C.

The concentrated anthocyanin extract was further refined by solid phase extraction (SPE) in C18 cartridges. The aqueous extract of anthocyanin was passed through a sorbent C18 poly-sery cartridge previously activated with methanol and equilibrated with water. The extract adsorbed onto the cartridge was rinsed with ultra-pure water to remove water-soluble impurities, and then eluted with acidified methanol (1% formic acid, *v*/*v*) [19]. Methanol was removed under vacuum at 35 °C and the residue was freeze-dried. Approximately 12 mg purified extract was obtained from 20 g of powdery Perilla leaves.

### 3.5. Total Anthocyanin Content (TAC)

As an important index of anthocyanins, monomeric anthocyanin pigment content was determined by the pH differential method described by [20]. The pigment concentrations were calculated by the following formula:

(1) Monomeric anthocyanin pigment (mg/L) = (A × MW × DF × 1000)/(ε×1)

Absorbance was calculated as A = (A_520_ − A_700_)_pH 1.0_ − (A_520_ − A_700_)_pH 4.5_ with a molar absorptivity (ε) of 26,900 (L·cm^−1^·mol^−1^) and molecular weight of 449.2 (g/mol). Anthocyanin content was mg/L which was then converted to mg/100 g dried sample. The data were expressed as the mean values ± SD (*n* = 3).

### 3.6. UPLC-ESI-IT-TOF-MS^n^ Analysis

Freeze dried samples were redissolved in a mixture of water/methanol/formic acid (80:20:1, v/v/v) and were analyzed by UPLC-ESI-IT-TOF-MS*^n^*. The final concentration of the sample was 0.1 mg/mL.

UPLC-ESI-IT-TOF-MS*^n^* was recorded on an UFLCXR system incorporating two LC-20ADXR pump, a SIL-20ACXR autosampler, a CTO-20AC column oven, a DGU-20A_5_ vacuum degasser, a SPD-M20A Uv-Vis detector and an LCMS-IT-TOF Mass Spectrometer (Shimadzu, Kyoto, Japan). Reversed phase separation was performed using a Shim-pack XR-ODS II column (2.0 × 100 mm, 2 μm) (Shimadzu, Kyoto, Japan) with a flow rate of 0.4 mL/min thermostated at 35 °C. Mobile phase A was 1% formic acid in water and mobile phase B was methanol. The gradient profile was as follows: solvent B increased from 20%–30% in the first 3 min, and then continually increased to 50% at 20 min, and finally reconditioned with 20% B for 5 min (total run time 25 min). The injection volume was 6 μL.

The MS parameters were as follows: positive ion mode was utilized by the ESI source. The full scan mass spectra covered the range from *m*/*z* 150–1000 Da (MS^1^). As for MS^2^ and MS^3^ the scan range was modified according to the precursor ions. Heat block and curved desolvation line temperatures were both 200 °C. Nebulizing nitrogen gas flow was 1.5 L/min, whilst the desolvation nitrogen gas flow was 10 L/min. The interface voltage was 4.5 kV for PI. The detector voltage was 1.60 kV. The relative collision induced dissociation energy was adjusted between 20%–30%. All data were recorded and processed by Shimadzu software LCMS solution version 3.70, Formula Predictor version 1.2 and Accurate Mass Calculator (Shimadzu, Kyoto, Japan).

For quantification, the peak areas of the isolated compounds were integrated from the HPLC chromatogram at 520 nm using LCMSsolution software. Malonyl-shisonin (**7**) was the most predominant anthocyanin (19.0 mg/100 g) followed by shisonin (**5**) (13.9 mg/g), malonyl-*cis*-shisonin (**4**) (9.3 mg/g), *cis*-shisonin (**1**) (4.4 mg/g), Cyanidin 3-*O*-caffeoylglucoside-5-*O*-glucoside (**2**) (3.0 mg/g), Cyanidin 3-*O*-caffeoylglucoside-5-*O*-malonylglucoside (**3**) (1.6 mg/g), Cyanidin 3-*O*-feruloylglucoside-5-*O*-glucoside (**6**) (1.3 mg/g). Each anthocyanin was quantified in cyanidin-3-*O*-glucoside equivalents (mg/100 g dry weight).

### 3.7. DAPI Fluorescence Staining

Purified anthocyanins were redissolved in sterilized water with a stock concentration of 1 mg/mL. Cells treated with different concentrations (0, 100, 150, 200, 250, 300 μg/mL) of anthocyanins were placed in a 5% CO_2_ incubator for 24 h at 37 °C. After the incubation procedure, the cells were fixed with 4% formaldehyde for 30 min and then washed three times. The stock solution of DAPI was diluted to a concentration of 10 μM and added to the cells. After 10 min staining at room temperature, unincorporated DAPI solution was removed. Cells were mounted with anti-fluorescence quenching liquid seal pieces, and then observed under a fluorescence microscope.

### 3.8. Annexin-V FITC/PI Flow Cytometric Analysis

The detection was performed utilizing the Annexin-V FITC/PI apoptosis detection kit. Briefly, cells (2 × 10^6^ cells/mL) treated with various concentrations (0, 200, 250, 300 μg/mL) of anthocyanin and cultured for 12 h were harvested, washed twice with cold PBS and resuspended with 100 μL binding buffer. Afterwards, 5 μL PI and Annexin V-FITC were added. The cells was incubated at 4 °C for 15 min in darkness and then analyzed for apoptosis on a BD FACSAria flow cytometer. Data were further evaluated by Cell Quest and FlowJo.

## 4. Conclusions

In our study, the characterization of anthocyanins in a rare Perilla variety cultivated in China was carried out by UPLC combined with DAD detector, TOF-MS and MS*^n^* analysis. The inherent advantages of high mass accuracy measurement and structural identification power utilizing the LCMS-IT-TOF instrument have showed us a new approach for the detailed investigation of anthocyanins in plant extracts. The developed approach involves a two-step analysis: in the first step, the EIC analysis combined with RP-LC chromatographic elution order allows tentative compound identification. Then, the structural elucidation by the second step MS*^n^* analysis ensures unambiguous identification of detected compounds. This methodology therefore offers a powerful and simple approach for the selective determination of anthocyanin derivatives. Seven anthocyanins were differentiated from *P. frutescens* var. *acuta*, most of which are consistent with the other varieties of *P. frutescens*. To our knowledge, the feruloylglycosylated anthocyanin was first reported in *P. frutescens*. Furthermore, this paper presents the first anticancer activity research on the anthocyanins in *P. frutescens*. The results showed that Perilla anthocyanins could induce Hela cell apoptosis. However, further studies are required to clarify the mechanism of action and the bioavailability of anthocyanins in Hela cells.

## Supplementary Materials

Supplementary materials can be accessed at: http://www.mdpi.com/1420-3049/20/05/9155/s1.
