# Supplementary material for: Characterization of Anthocyanins in Perilla frutescens var. acuta Extract by Advanced UPLC-ESI-IT-TOF-MSn Method and Their Anticancer Bioactivity
Source: Molecules. 2015 May 19;20(5):9155–69. doi: 10.3390/molecules20059155 (PMC6272396; doi:10.3390/molecules20059155)
Supplement: Supplementary file 1 [file molecules-20-09155-s001.pdf]

Supplementary Materials

1. Cis-shisonin

$[M]^+$ *m/z* 757.1965
molecular formula: $C_{36}H_{37}O_{18}^+$
MS[1]

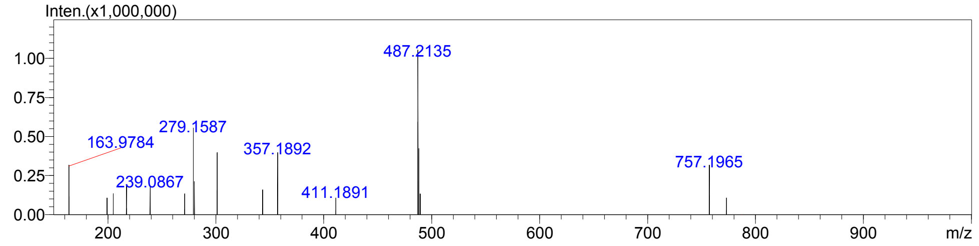

MS[2] precursor ion 757.1965

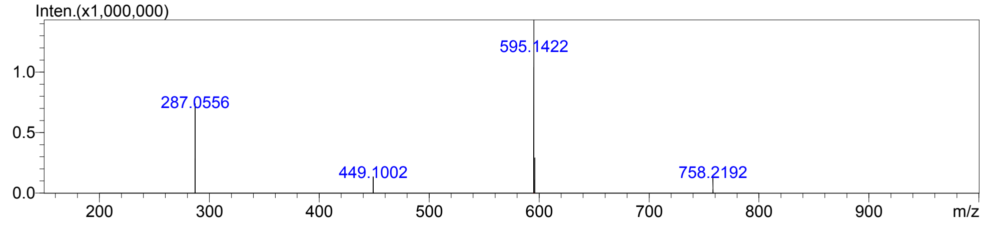

MS[3] precursor ion 595.1422

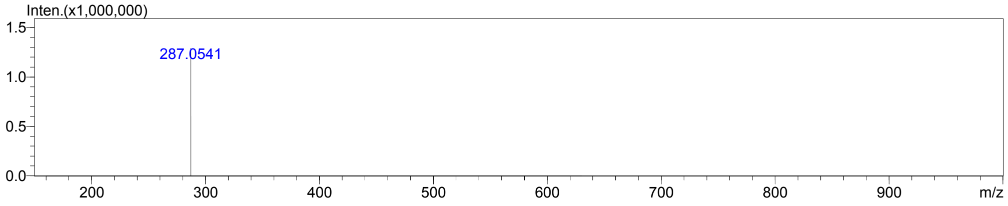

Uv-Vis 250–800 nm

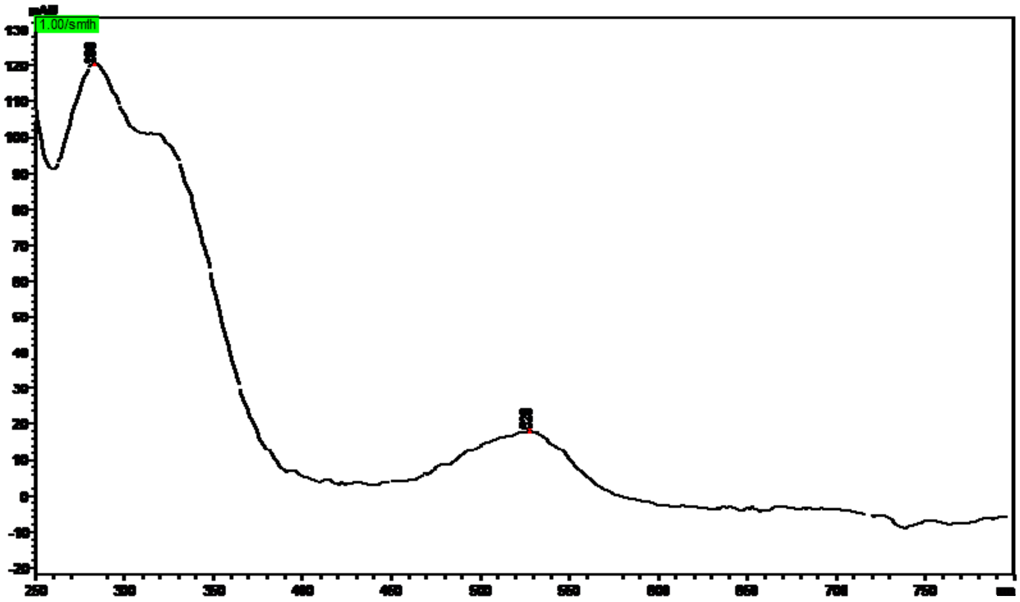

2. Cyanidin 3-O-caffeoylglucoside-5-O-glucoside

$[M]^+$ *m/z* 773.1932
molecular formula: $C_{36}H_{37}O_{19}^+$
MS^1

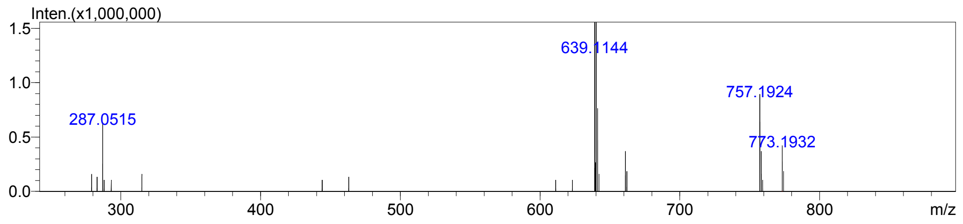

MS^2 precursor ion 773.1932

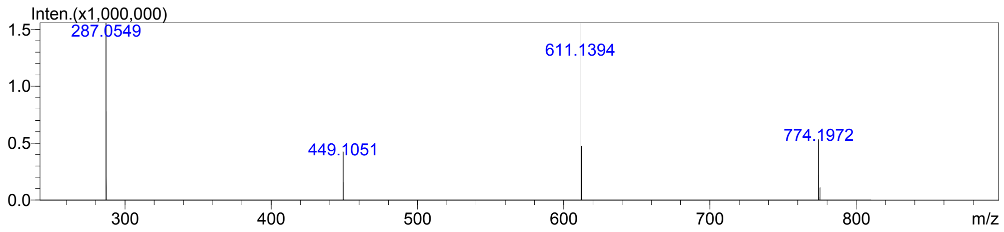

MS^3 precursor ion 611.1394

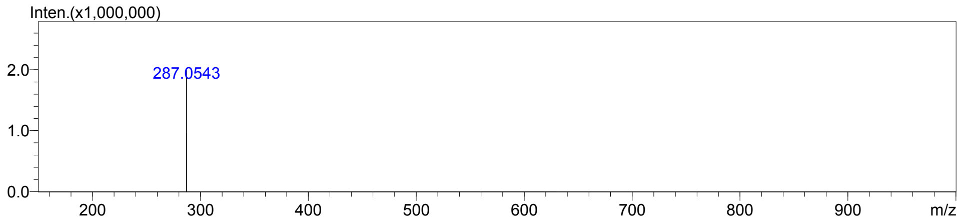

Uv-Vis 250–800 nm

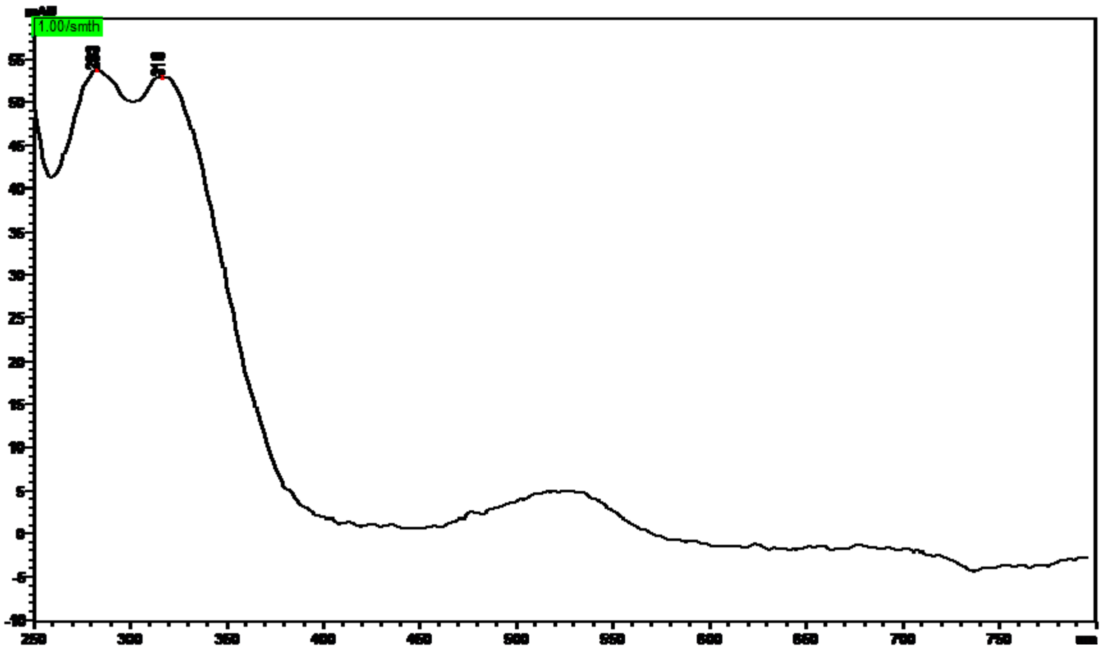

3. Cyanidin 3-O-caffeoylglucoside-5-O-malonylglucoside

[M]$^{+}$ *m/z* 859.1926

molecular formula: $C_{39}H_{39}O_{22}^{+}$

MS^{1}

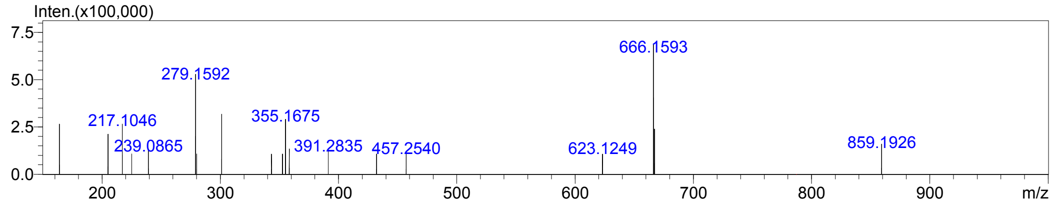

MS^{2} precursor ion 859.1926

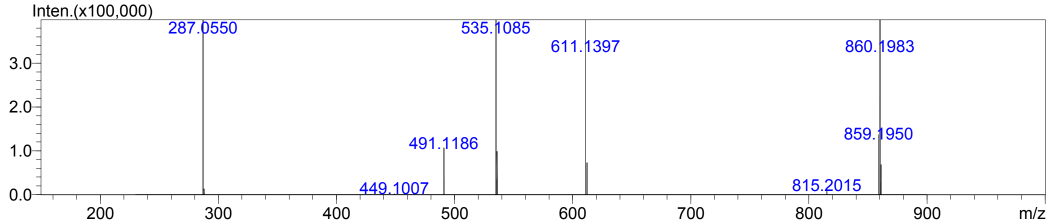

MS^{3} precursor ion 535.1085

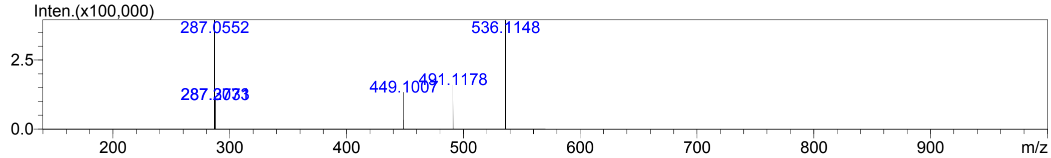

Uv-Vis 250–800 nm

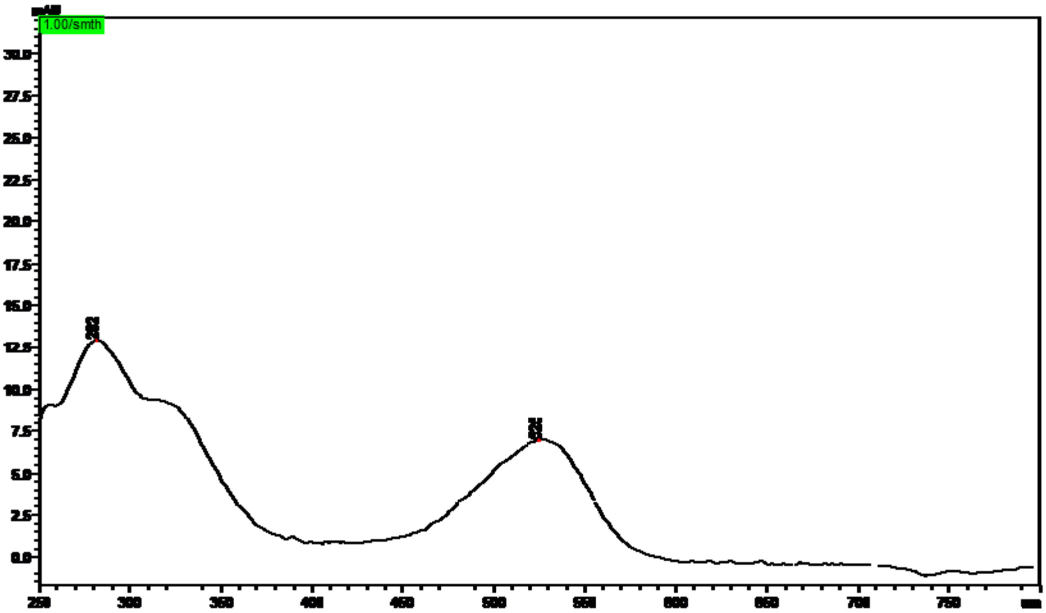

4. Malonyl-cis-shisonin

$[M]^+$ *m/z* 843.1965
molecular formula: $C_{39}H_{39}O_{21}^+$
MS^1

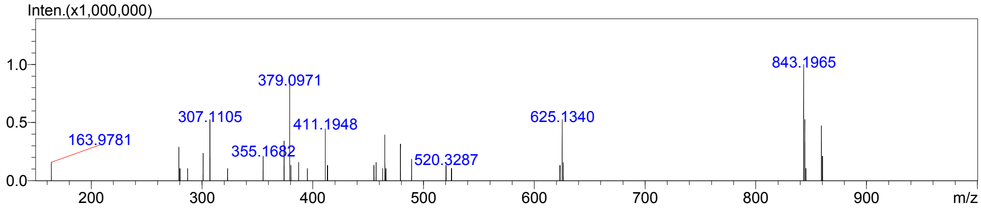

MS^2 precursor ion 843.1965

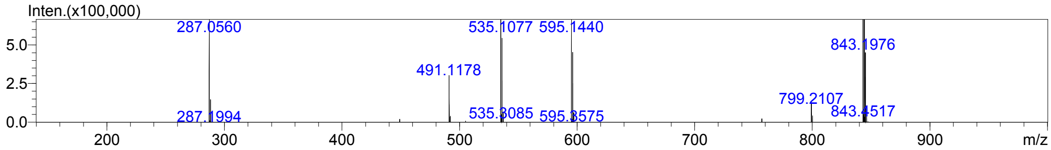

MS^3 precursor ion 535.1077

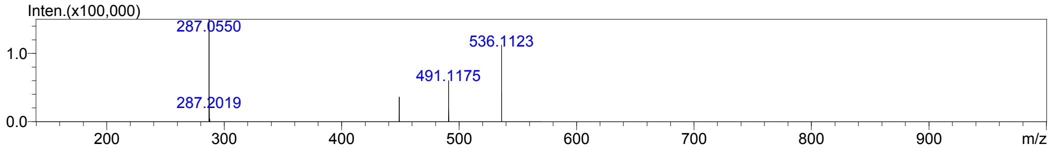

MS^3 precursor ion 595.1440

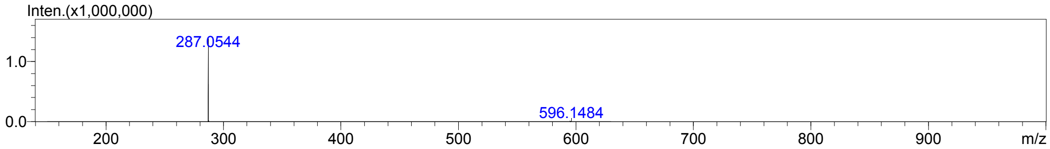

Uv-Vis 250–800 nm

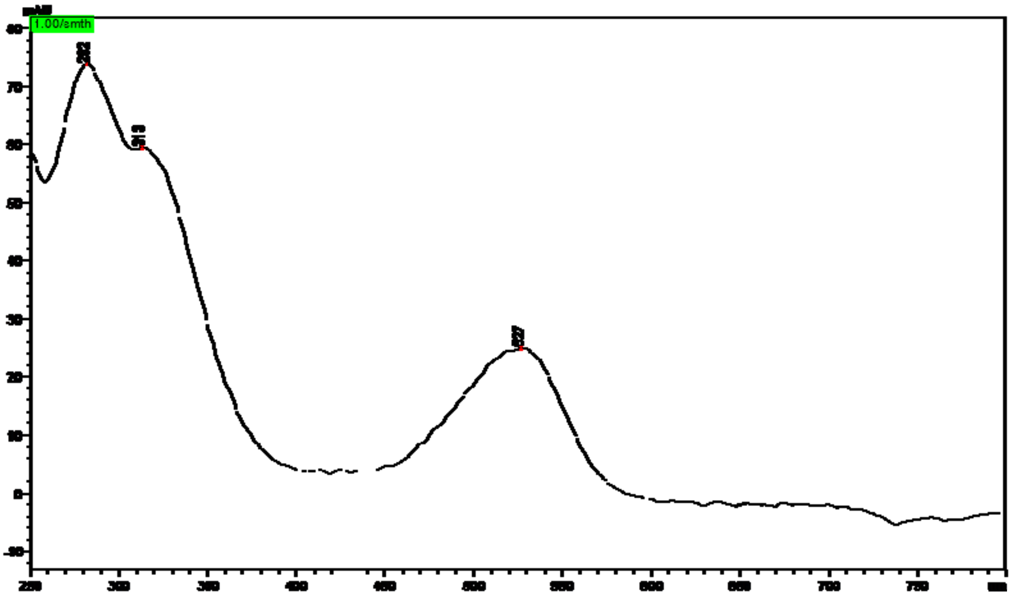

5. Shisonin

$[M]^+$ *m/z* 757.1973

molecular formula: $C_{36}H_{37}O_{18}^+$

MS^1

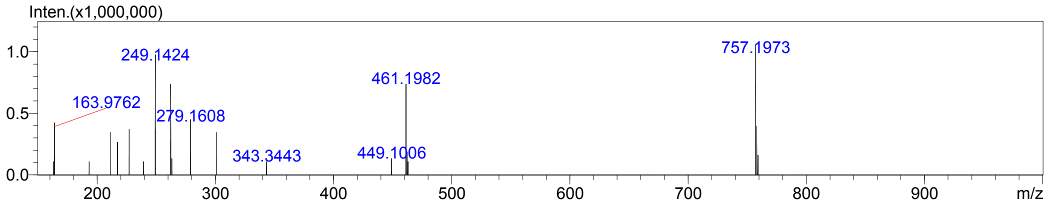

MS^2 precursor ion 757.1973

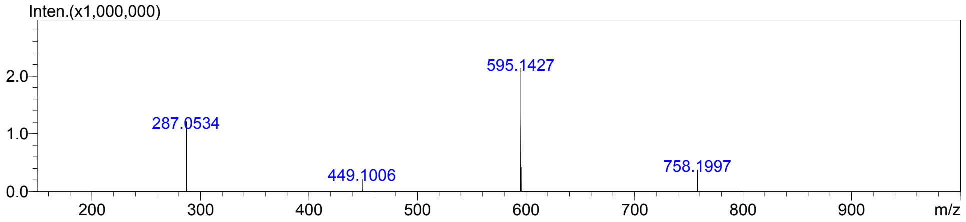

MS^3 precursor ion 595.1427

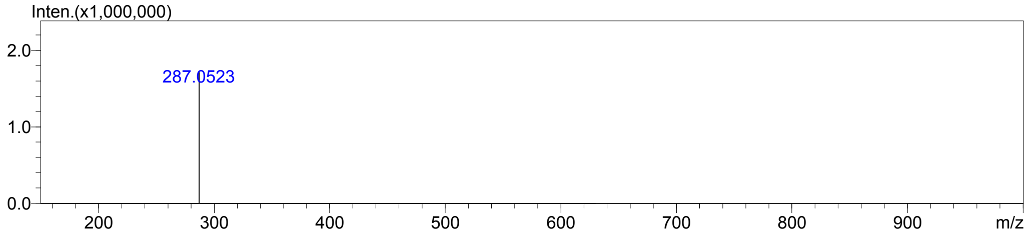

Uv-Vis 250–800 nm

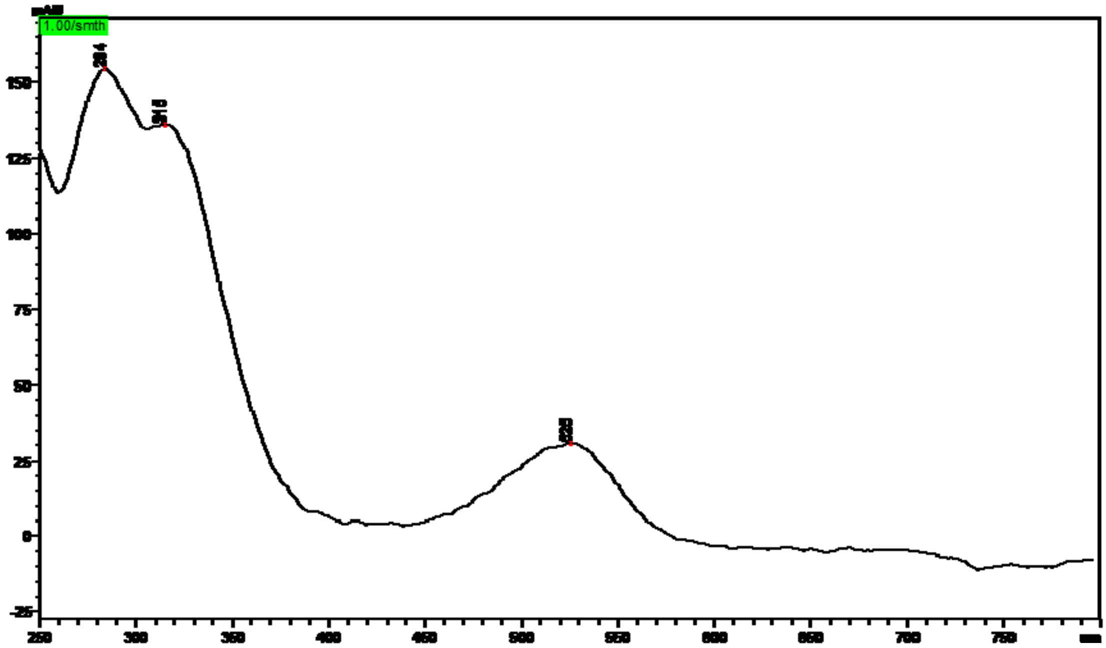

6. Cyanidin 3-O-feruloylglucoside-5-O-glucoside

[M]+ *m*/*z* 787.2097

molecular formula: C37H39O19+

MS[1]

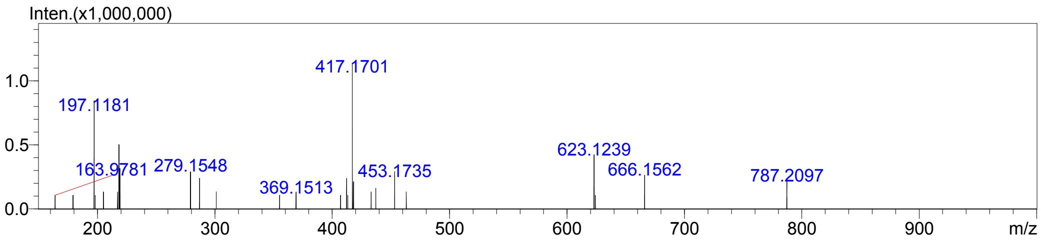

MS[2] precursor ion 787.2097

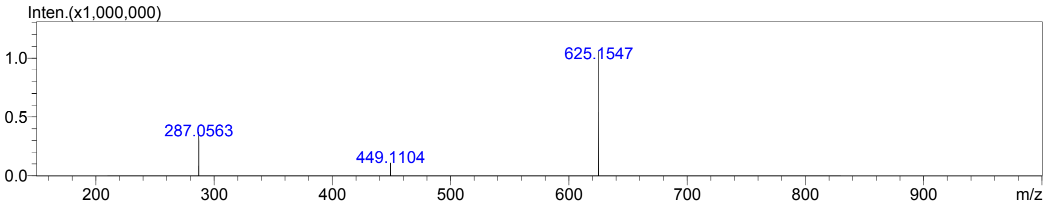

MS[3] precursor ion 625.1547

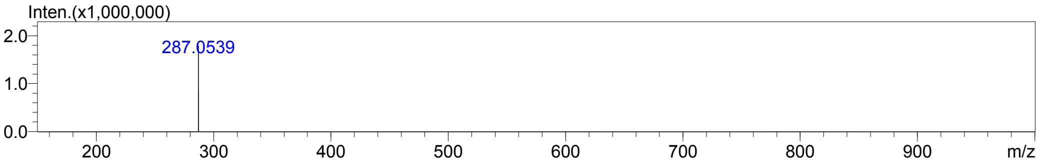

Uv-Vis 250–800 nm

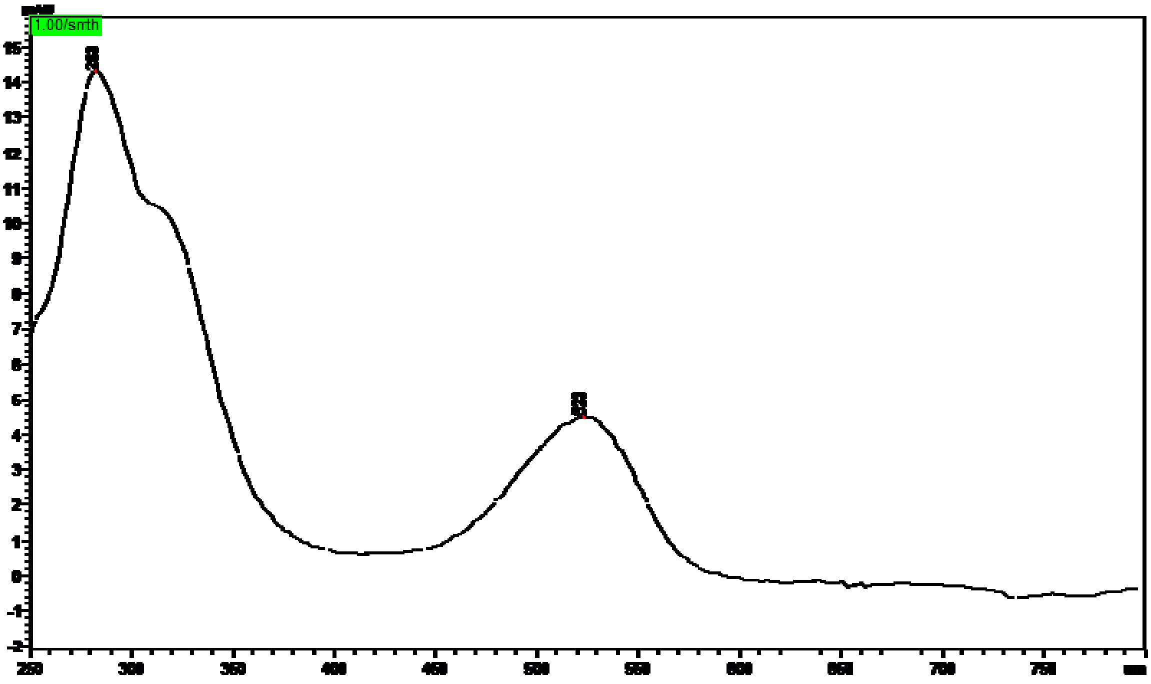

7. Malonyl-shisonin

$[M]^+$ *m/z* 843.1952

molecular formula: $C_{39}H_{39}O_{21}^+$

MS^1

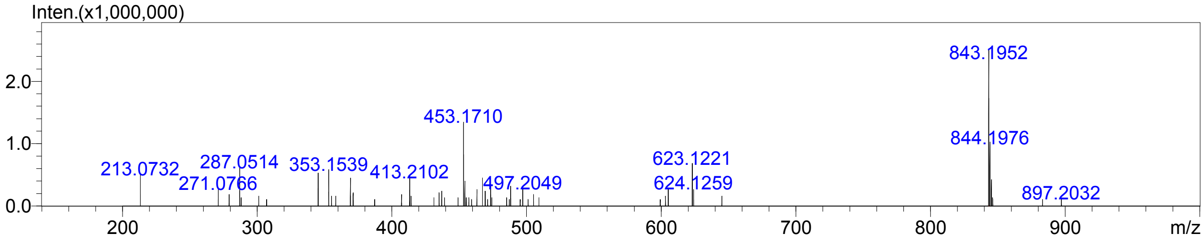

MS^2 precursor ion 843.1952

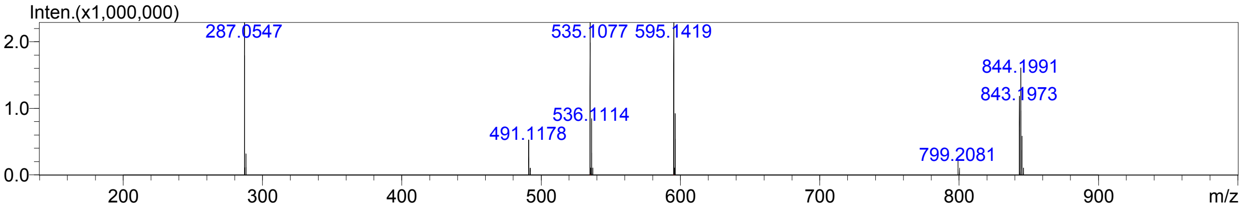

MS^3 precursor ion 535.1077

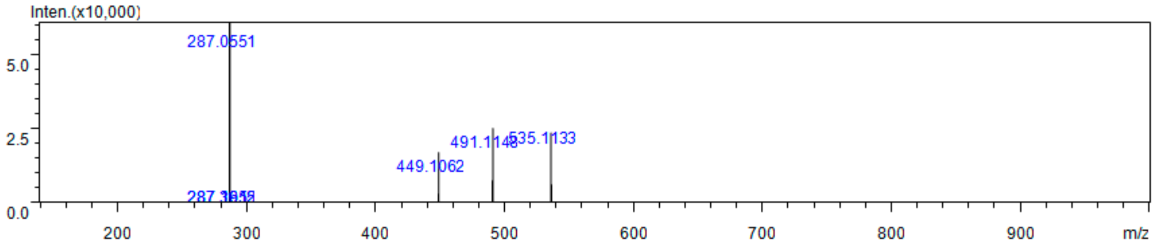

MS^3 precursor ion 595.1419

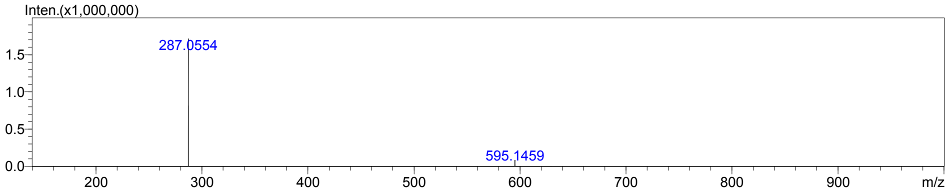

Uv-Vis 250–800 nm

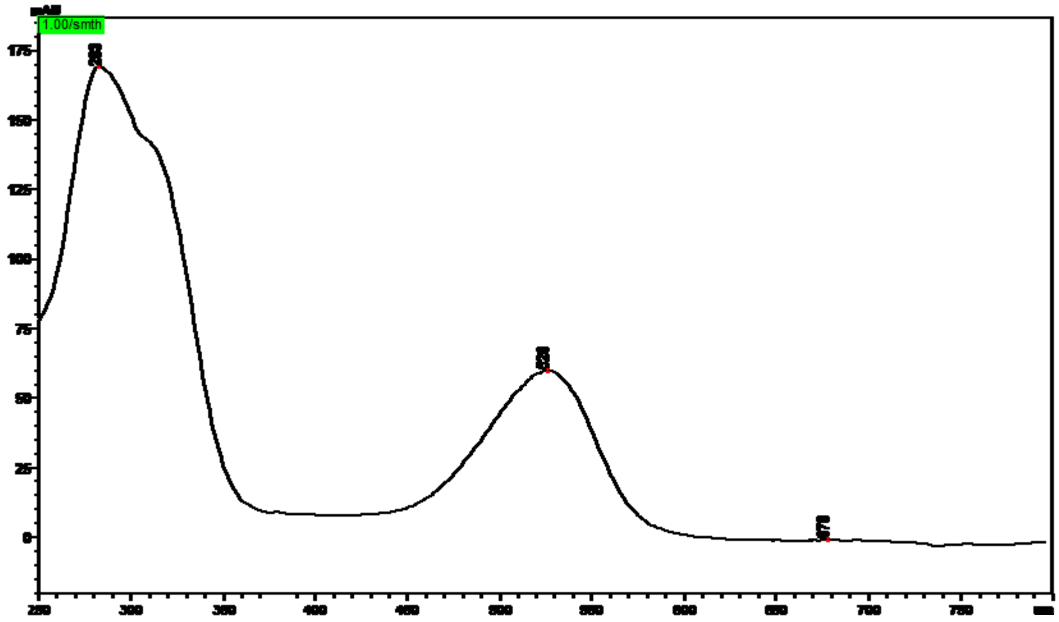

Formula Predictor (isotope pattern comparison)

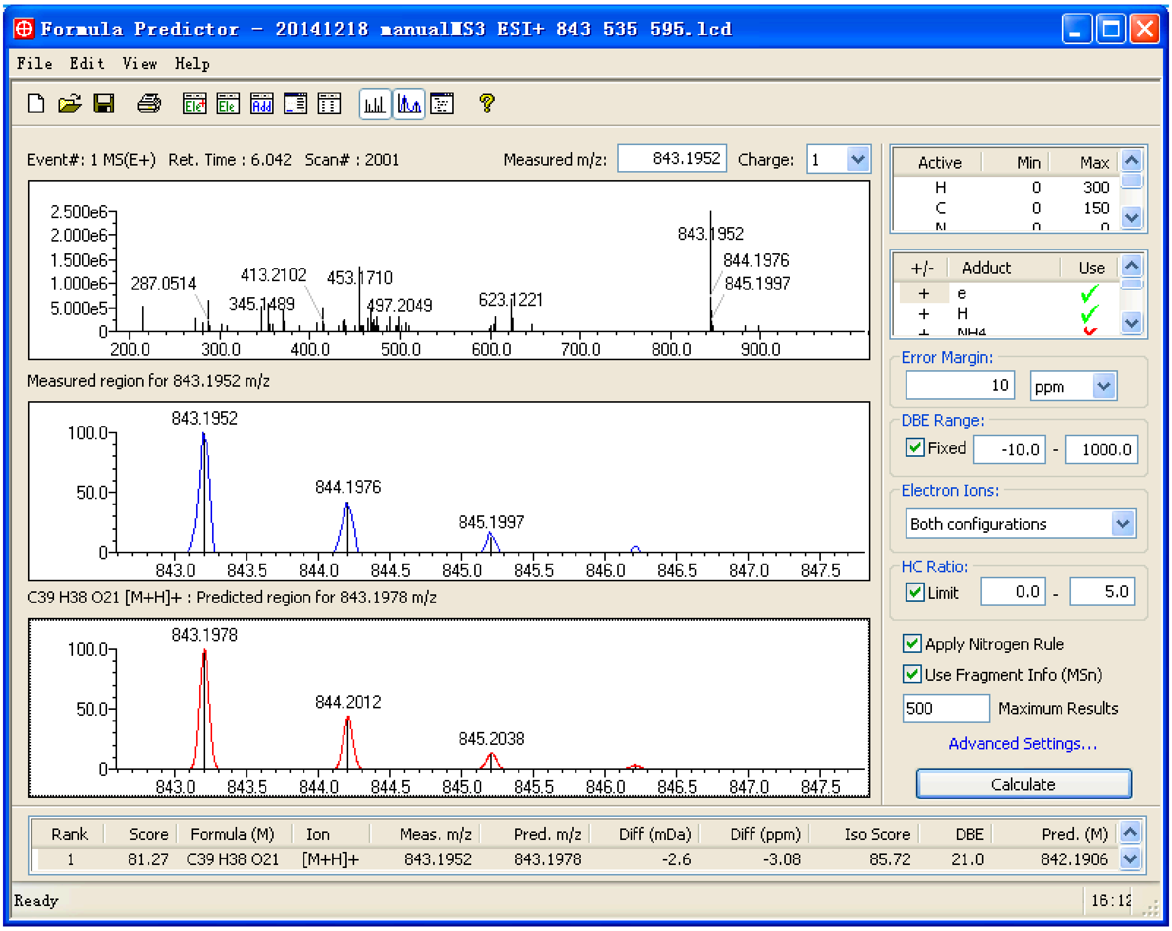

Accurate Mass Calculator (neutral loss calculation)

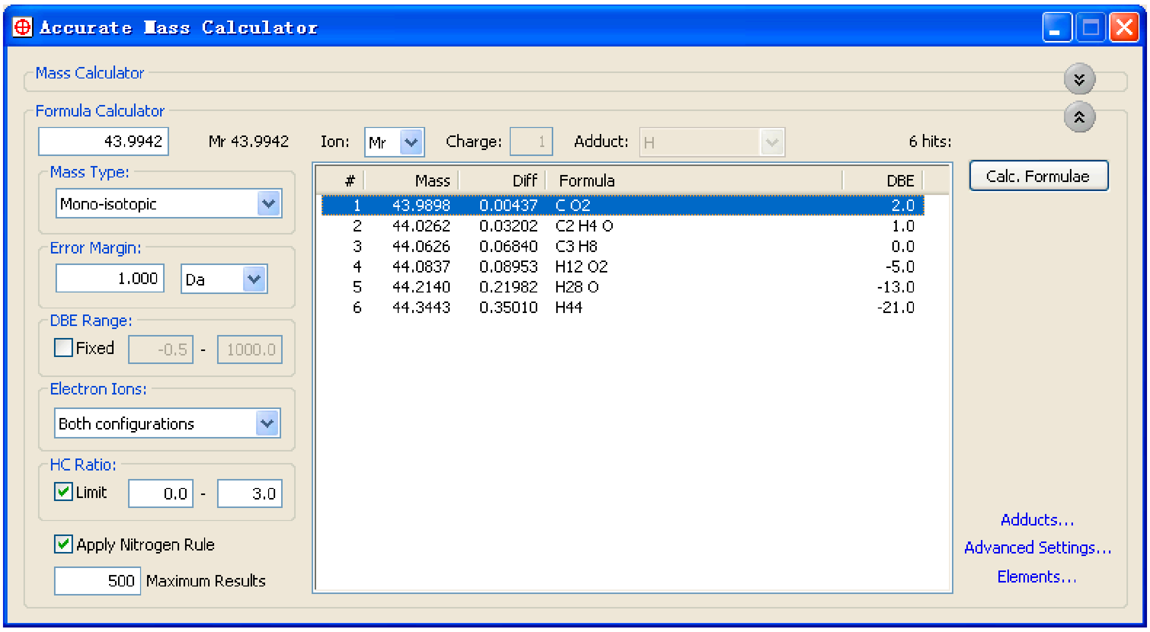

The choice of concentration was justified according to a previous anti-proliferation assay using a modified MTT assay (CCK-8 method).

Method:

The effect of anthocyanins on cell proliferation was determined using the CCK-8 assay, which has a higher sensitivity than other traditional proliferation analysis such as MTT or XTT. Briefly, Hela cells with a concentration of 5×10^4 cell/mL were seeded into a 96-well plate (5×10^3 cells for each well), which was placed in the 5% CO_2 incubator for 24 h at 37 °C. The cells were then treated with different concentrations of anthocyanins (50, 100, 150, 200, 250, 300 $\mu g \cdot mL^{-1}$) for 12 h. At the end of the incubation, 10 μL CCK-8 was added in each well, which was cultured for 2 h in the incubator and then the absorbance was measured with a microplate reader at 450 nm wavelength to calculate the inhibition rate.

Results:

The proliferation assay was performed by a modified MTT assay (CCK-8 method) testing, and the result showed that cell viability in the model group was obviously lower than that of control group, indicating Perilla Anthocyanin has inhibited Hela cell proliferation with evident dose-dependency (Figure 1). The IC_{50} (12 h) value of 253.4 $\mu g \cdot mL^{-1}$ was obtained by SPSS simulation.

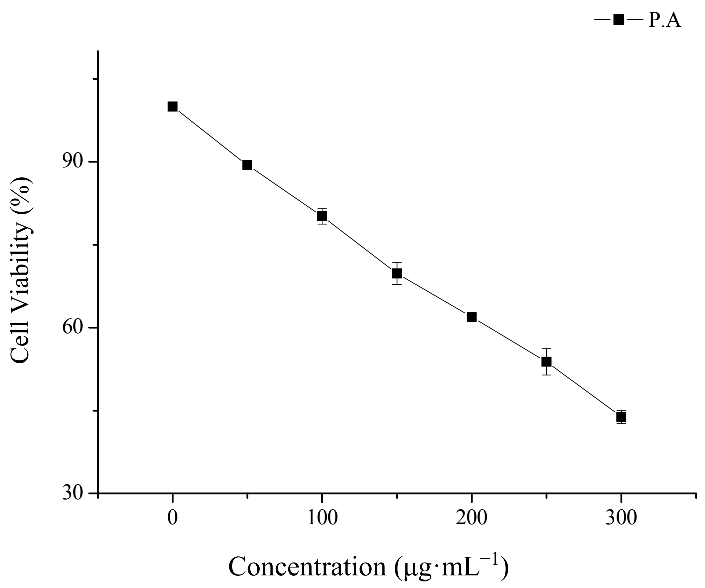

Figure S1. Effects of Perilla anthocyanins on Cell proliferation; Data are mean ± SD of three independent experiments; Values are expressed in percentage and referred to control cells.

The concentrations of apoptosis was chosen on the basis of the IC_{50} value. Concentrations around 253.4 μg·mL^{-1} was tested in the following apoptosis experiment.

DAPI Fluorescence Staining with different concentrations.

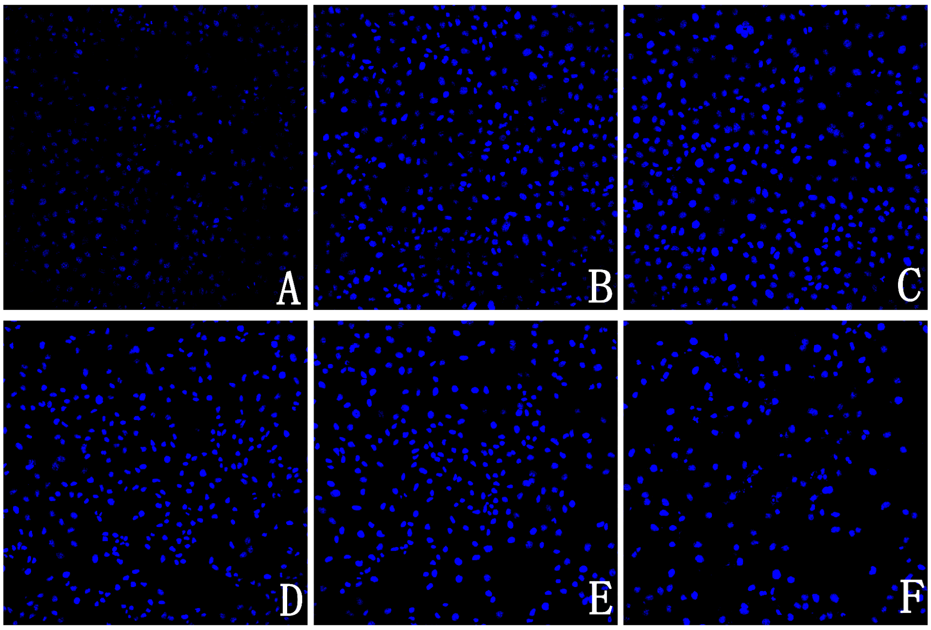

Figure S2. Laser scanning confocal microscope (200×) results of Perilla anthocyanin treated cells (**A**) 0 μg·mL^{-1}; (**B**) 100 μg·mL^{-1}; (**C**) 150 μg·mL^{-1}; (**D**) 200 μg·mL^{-1}; (**E**) 250 μg·mL^{-1}; and (**F**) 300 μg·mL^{-1}.

Typical apoptosis morphology was shown in the main article (Figure 5).
